# Immune Reconstitution in Pediatric Aplastic Anemia after Allogeneic Hematopoietic Stem-cell Transplantation

**DOI:** 10.7150/ijms.70146

**Published:** 2022-05-01

**Authors:** Jiayu Wang, Meng Yuan, Guanghua Zhu, Runhui Wu, Chenguang Jia, Bin Wang, Jie Zheng, Jie Ma, Maoquan Qin, Sidan Li

**Affiliations:** 1 Hematology Center, Beijing Key Laboratory of Pediatric Hematology Oncology; National Key Discipline of Pediatrics (Capital Medical University); Key Laboratory of Major Diseases in Children, Ministry of Education; Beijing Children's Hospital, Capital Medical University, National Center for Children's Health, China; 2 Department of Medical Oncology, National Cancer Center/National Clinical Research Center for Cancer/Cancer Hospital, Chinese Academy of Medical Sciences and Peking Union Medical College, Beijing, China

**Keywords:** aplastic anemia, allogeneic hematopoietic stem-cell transplantation, immune reconstitution, pediatric

## Abstract

**Background:** Previous studies had revealed that immune reconstitution (IR) after allogeneic hematopoietic stem-cell transplantation (allo-HSCT) affected the clinical prognosis of patients. However, few studies were based on pediatric patients and patients with aplastic anemia (AA). The purpose of this research was to analyze IR of pediatric AA after HSCT and further explore its clinical prognostic value.

**Methods:** The whole of 61 pediatric patients with AA who underwent HSCT were enrolled. Lymphocyte subsets count in peripheral blood, CD4^+^/CD8^+^ T cell ratio, and serum concentration of immunoglobulins were detected using flow cytometry at regular intervals after HSCT.

**Results:** Innate immunity recovered faster than adaptive immunity, T lymphocytes recovered faster than B lymphocytes. The number of transfused CD34^+^ cells and the implantation time of ANC significantly affected the early rapid IR of CD3^+^ T cells. The degree of HLA site coincidence significantly affected the early rapid IR of CD19^+^ B cells. The number of transfused MNC and CD34^+^ cells significantly affected the early rapid IR of CD56^+^ NK cells. The overall survival (OS) and failure-free survival (FFS) of CD56^+^ NK cells in early rapid IR group were higher than those in non-IR group. The CD3^+^ T cell early rapid IR group and CD8^+^ T cell early rapid IR group had higher OS than the non-IR group.

**Conclusion:** Early rapid IR after HSCT is a good predictor of clinical prognosis in children with AA. This study provides a reasonable prediction for early rapid IR, which may improve clinical outcomes of children.

## Introduction

For aplastic anemia (AA), especially severe AA (SAA), allogeneic hematopoietic stem-cell transplantation (allo-HSCT) is a potentially curative therapy to save the life of patients by reconstituting hematopoietic and immunologic systems. However, allo-HSCT is accompanied by certain risks, such as infection, acute and chronic graft-versus-host disease (GVHD). Several studies had shown that the rapidity and degree of immune reconstitution (IR) were closely related to the occurrence, development and outcome of transplantation complications, and ultimately affected the overall survival (OS) 1, 2.

Compared to patients with malignant hematological diseases, the rapidity of IR in patients with AA after transplantation is relatively slow 3. Currently, most studies of IR are based on malignant hematological diseases, or only include a small sample of adult patients with AA. Therefore, in this study, we investigated IR after HSCT in children with AA, and provided certain insights into the association between IR and clinical prognosis.

## Methods

### Clinical materials

We retrospectively analyzed the clinical data of 61 AA children who underwent HSCT in Beijing Children's Hospital from February 2008 to October 2020. The diagnosis and typing of AA would have to be based on international standardization of guidelines for the diagnosis and treatment 4. All results of the chromosome breakage assay were negative. No dyskeratosis congenital was included. Patients with clonal evolution were excluded from this study. Signed informed consent forms were obtained from all guardians of children, the diagnosis and treatment plans were also approved by the Ethics Committee of Beijing Children's hospital. These patients were aged at 1 year 2 months to 16 years 9 months with a median age of 7 years 6 months (90 months). Of these patients, 29 were boys, 32 were girls. There were 21 cases of matched related donor HSCT (MRD-HSCT), 10 cases of matched unrelated donor HSCT (MUD-HSCT), and 30 cases of haploidentical HSCT (Haplo-HSCT). Most patients had received multiple infusions of blood products and 7 children had been treated with anti-thymocyte globulin (ATG). The general clinical characteristics are shown in Table 1.

### Conditioning regimens

The basic regimen of 50 mg/kg/d cyclophosphamide (Cy) and 3 mg/kg ATG or Cy and 5 mg/kg antilymphocyte globulin (ALG) was used to treat 9 patients at -5 to -2 days before transplantation (CA). Besides the basic regimen, 36 patients were also treated with 25 mg/m^2^/d fludarabine (Flu) at -6 to -2 days to enhance the immunosuppressive effect (FCA). Based on the FCA conditioning regimen, 6 patients were given 3 Gy TBI (TBI+FCA) and 9 patients were added with 0.8 mg/kg busulfan (Bu) every 6 hours at -7 and -6 days (Bu+FCA) to eliminate the residual hematopoiesis. There was 1 patient with chromosomal abnormalities, who was treated with 2 g/m^2^ arabinoside (Ara-C) every 12 hours at -6 and -5 days (Ara-C+FCA).

### Mobilization, collection and reinfusion of hematopoietic stem cells

To mobilize donor stem cells, we used 5~10 μg/kg of granulocyte colony-stimulating factor (G-CSF) continuously for 5 days. Donor bone marrow (BM) was collected on day 4 and peripheral blood stem cells (PBSC) on day 5. If neither the total number of mononuclear cells (MNC) nor the number of CD34^+^ cells was meeting the standard of transplantation on day 5, the collection was repeated on day 6. Red blood cells were removed using hydroxyethyl starch if the donor and recipient had incompatible major ABO blood types, and plasma was removed using density gradient centrifugation if the donor and recipient had incompatible minor ABO blood types.

### Prevention of GVHD

Acute and chronic GVHD were evaluated by modified Glucksberg grading system 5 and Chinese consensus on the diagnosis and management of chronic graft-versus-host disease (2021) 6 respectively. The basic GVHD prophylaxis regimens consist of cyclosporin A (CsA) or CsA+short-term methotrexate (stMTX) or CsA+stMTX+mycophenolate mofetil (MMF). We treated 15 cases of MRD and 2 cases of Haplo with CsA only. We used CsA+stMTX to prevent GVHD in 6 cases of MRD, 1 case of MUD and 9 cases of Haplo. We treated 9 cases of MUD and 19 cases of Haplo with CsA+stMTX+MMF to enhance inhibition of T and B cell proliferation. Patients who developed Ⅱ~Ⅳ° aGVHD received methylprednisolone (a total of 1~2 mg/kg) or anti-CD25 monoclonal antibody. If patients had stable donor chimerism and no obvious GVHD, CsA and MMF were tapered during 6 months. After HSCT, 24 children received mesenchymal stem cells (MSCs) infusion to prevent GVHD.

### Detection of indicators related immune reconstitution

The counts of CD3^+^ T, CD4^+^ T, CD8^+^ T, CD19^+^ B, CD56^+^ NK cells, CD4^+^/CD8^+^ T cell ratio in peripheral blood were detected by flow cytometry and the quantification of immunoglobulin IgA, IgE, IgG, IgM were detected by immunoturbidimetry at 1, 2, 3, 6, 9, 12, 18, 24, 36 months after transplantation. The thresholds defined early rapid IR for CD3^+^ T, CD4^+^ T, CD8^+^ T, CD19^+^ B, CD56^+^ NK were 700 /uL, 200 /uL, 500 /uL, 50 /uL and 100 /uL in the 3rd month after HSCT, respectively 7.

### Follow-up

The median follow-up time was 38 months (3-115 months). All surviving patients were followed up for more than 6 months. OS was defined as the survival time from diagnosis to death or the last clinical follow-up. Failure-free survival (FFS) was defined as the survival time from diagnosis to the emergence of transfusion-dependent events 8.

### Statistical analysis

SPSS20.0 software was used for data processing and statistical analysis. P≤0.05 was considered statistically significant. The median and 25th-75th percentile of immune cell subsets and immunoglobulin quantification at 1, 2, 3, 6, 9, 12, 18, 24, 36 months after transplantation were statistically analyzed to describe IR. For categorical variables, the univariate analysis was performed using binary logistic regression analysis or chi-square test. For continuous variables, two independent sample t-test or nonparametric rank-sum test were applied. Factors with statistical significance were further entered into multivariate analysis using binary logistic regression analysis. Kaplan-Meier method was used for survival analysis. P value was calculated by log-rank test.

## Results

### IR characteristics

**Figure 1:**
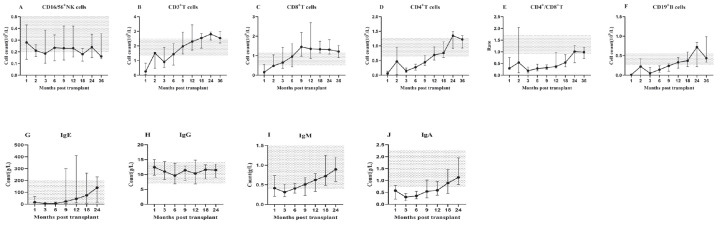
IR in lymphocyte subsets (A-F): The median counts and 25th-75^th^ percentiles of immune cell subsets. (G-J): The median counts and 25th-75th percentiles of immunoglobulin quantification. Error bars indicate the 25th to 75th percentiles. Shaded areas represent the normal range.

The count of immune cell subsets, CD4^+^/CD8^+^ T cell ratio and immunoglobulin quantification at 1, 2, 3, 6, 9, 12, 18, 24, 36 months after transplantation were monitored. The median and 25th-75th percentile was shown in Figure 1.

After HSCT, innate immune CD56^+^ NK cells first recovered, rapidly reached the normal level in the 1st month and remained within the normal range (Figure 1A). Adaptive immune T lymphocytes recovered before B lymphocytes. CD3^+^ T cells were close to the normal level in the 2nd month and remained stable (Figure 1B). Among T cell subsets, CD8^+^ cytotoxic T cells recovered significantly faster than CD4^+^ helper T cells. CD8^+^ T cells remained above the normal level in the 2nd month (Figure 1C), while CD4^+^ T cells remained below the normal level until 12 months after transplantation, and recovered slowly in the first 2 years (Figure 1D). These findings uncovered the underline reason for CD4^+^/CD8^+^ T cell ratio inversion in the first 2 years after transplantation. Based on our observation, the ratio reached the normal range in the 24th month (Figure 1E). However, CD19^+^ B cells remained below the normal level for about 1 year after transplantation, especially almost disappeared in the first 3 months, and then began to recover slowly, but the recovery was delayed compared to other subsets. The normal level was not reached until the 2nd year after transplantation (Figure 1F).

The recovery of immunoglobulin also had its characteristics. The concentrations of IgE (Figure 1G), IgG (Figure 1H) and IgM (Figure 1I) showed a certain degree of fluctuation, while, within the normal range. In contrast, the concentration of IgA (Figure 1J) kept a low level in the first 12 months and returned to normal in the 2nd year after HSCT.

### Factors affecting early rapid IR

In this study, we monitored 61 children for early rapid IR in the 3rd month after HSCT. Among them, 57.4% patients (35 cases) reached CD3^+^ T cell IR, 26.2% patients (16 cases) reached CD4^+^ T cell IR, 59.0% patients (36 cases) reached CD8^+^ T cell IR, 45.9% patients (28 cases) reached CD19^+^ B cell IR and 72.1% patients (44 cases) reached CD56^+^ NK cell IR.

The clinical variables included in univariable analysis were gender, month age, ANC/HGB/PLT/RET level at the initial diagnosis, whether ATG was applied, whether infection occurred before transplantation, time from diagnosis to HSCT, graft type, stem cell source, whether HLA was matched, whether the blood types of donor and recipient were matched, whether TBI was applied, conditioning regimens, GVHD prophylaxis regimens, the number of transfused MNC, the number of transfused CD34^+^ cells, whether MSC was received, the implantation time of ANC, the implantation time of PLT, donor cell chimerism rate, whether aGVHD, cGVHD, CMV infection and EBV infection occurred after transplantation. Results suggested that the early rapid IR of CD56^+^ NK cells was affected by the number of MNC (P=0.050) and CD34^+^ cells (P=0.033). The early rapid IR of CD3^+^ T cells was affected by whether there was infection before transplantation (P=0.034), the number of transfused CD34^+^ cells (P=0.022) and the implantation time of ANC (P=0.021). The early rapid IR of CD4^+^ T cells was affected by the number of transfused CD34^+^ cells (P=0.04). Whether HLA was completely matched (P=0.002), whether MSC was received (P=0.038), whether aGVHD occurred (P=0.016) and graft type (P=0.020/0.036/0.009) influenced the early rapid IR of CD19^+^ B cells. In the present study, no significant factors were found in the early rapid IR of CD8^+^ T cells. Data are shown in Table 2.

Multivariate analysis suggested that the number of MNC (P=0.041) and CD34^+^ cells (P=0.024) significantly affected the early rapid IR of CD56^+^ NK cells. The number of transfused CD34^+^ cells (P=0.015) and the implantation time of ANC (P=0.027) significantly affected the early rapid IR of CD3^+^ T cells. As to the CD19^+^ B cells, children with HLA completely matched were more likely to receive IR than those with HLA incompletely matched (P=0.045). See Table 2.

### Early rapid IR and outcomes

The primary implantation failure was found in 2 patients and secondary implantation failure in 4 patients. Among the 61 children, 53 received stable implantation and survived. The 2 and 5-year cumulative OS rates were 91.5% and 87.4%, respectively. Among the 61 children, 11 had FFS events. The 2 and 5-year cumulative FFS rates were 91.5% and 83.5%, respectively.

**Figure 2:**
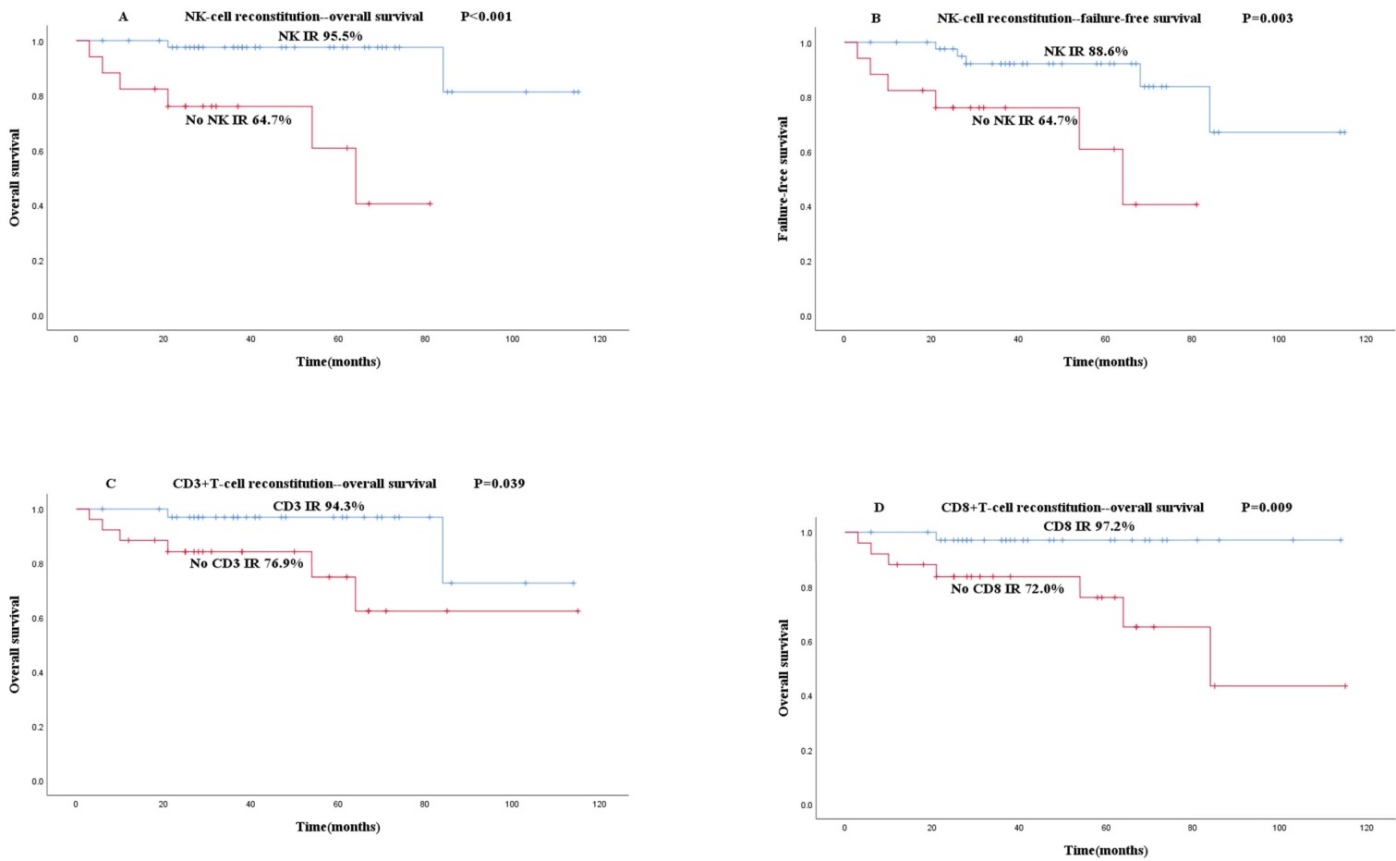
Association between early rapid IR and survival (A-D): The results of patients' OS and FFS are shown based on the IR results. Blue and red lines show patients with early rapid IR or not. (A) OS according to NK IR. (B) FFS according to NK IR. (C) OS according to CD3^+^T IR. (D) OS according to CD8^+^T IR.

To explore the relationship between early rapid IR and OS or FFS, the Kaplan-Meier curves of IR and non-IR groups were plotted and survival rates were analyzed. Compared with the CD56^+^ NK cell early rapid IR group, the OS (95.5% vs 64.7%, P<0.001, Figure 2A) and FFS (88.6% vs 63.7%, P=0.003, Figure 2B) of the non-IR group were both significantly lower. CD3^+^ T cell (94.3% vs 76.9%, P=0.039, Figure 2C) and CD8^+^ T cell early rapid IR group (97.2% vs 72.0%, P=0.009, Figure 2D) showed significantly higher OS than the non-IR group, independent of FFS. Early rapid IR of CD19^+^ B cells and CD4^+^ T cells were not associated with OS and FFS based on our research.

## Discussion

IR after HSCT is a complex step-by-step process. The recovery of immune cells and the reconstitution of immune system have a close relationship with the patient's post-transplantation complications, such as infection and GVHD, finally affecting the patient's outcome 1, 2. In this study, we retrospectively analyzed the IR of 61 pediatric patients with AA at different time points after transplantation.

Our results indicated that innate immune CD56^+^ NK cells, adaptive immune CD3^+^ T cells and CD19^+^ B cells were reconstituted sequentially. Among the CD3^+^ T cell subsets, CD8^+^ T cells recovered rapidly and reached the normal level in the 2nd month after transplantation, while the lack of CD4^+^ T cells would exceed 12 months. The CD4^+^/CD8^+^ T cell ratio was inverted in the first two years after transplantation, which shared same points with previous AA reports 9-11, and may be closely related to the ATG therapy before transplantation 12. In the current study, the reconstitution of immune cells was faster in patients with AA than in previous studies of transplant patients excluding AA 7. However, due to the diversity of graft types, stem cell sources and conditioning regimens in different centers, IR patterns are of individual features to some extent.

The lower level of immunoglobulin after HSCT reflects the absence of immunoglobulin-producing B cells 13. Our research showed that the recovery of IgA took more than one year and was basically synchronized with the reconstitution of CD19^+^ B cells. However, Ngwube et al. 10 found that the serum concentration of IgM was below the normal range on the 100th day. Pei et al. 9 found that the serum concentrations of IgM and IgA decreased significantly and did not return to normal by 365 days. Liang et al. 3 found that the serum concentrations of IgM and IgG were basically normal in the 6th month after transplantation, while the recovery of IgA took at least 1 year. These differences may be related to the diversity of conditioning regimens^14^. Another possible reason is that children after transplantation will receive interval immunoglobulin infusion to prevent virus infection, which may be a replenishment. In the future, new precise methods to distinguish Ig originations may help to draw a more reasonable conclusion.

Previous researches were focused on the IR of malignant hematological diseases, and few studies were focused on the IR of AA. More importantly, no pediatric data had been reported separately. Next, we tried to analyze the factors affecting early rapid IR and evaluate the predictive value of IR. As we know, thymus is crucial for the reconstitution of T cells 15. The function of thymus in children is better than that in adults. The study reported that younger recipient age was associated with better reconstitution of B cells owing to a potential role of non-hematopoietic/microenvironmental factors 16. It is necessary to explore early rapid IR in children with AA. Although the thresholds of early rapid IR used in this study were from the data of malignant hematological diseases 7, we found that they were also reasonable in patients with AA.

Numerous studies had shown that reinfusion of high-dose CD34^+^ cells led to promote the rapid implantation of neutrophils and platelets, but the specific value of this high-dose varied between studies 17, 18. Remberger et al. 19 recommended that the number of CD34^+^ cells be controlled between 2.5 and 11×10^6^/kg. Reinfusion of high-dose MNC could also promote the rapid implantation of neutrophils and platelets, when the dose was between 3 and 5.99×10^8^/kg was positively correlated with hematopoietic reconstitution 20. These researches included adult patients with malignant hematological diseases and did not directly investigate IR. Rapid and stable hematopoietic reconstitution is the most basic and critical step of IR. Our data showed that the implantation time of ANC was related to the early rapid IR of CD3^+^ T cells. The experimental model demonstrated that the reinfusion of more stem cells was associated with the rapid reconstitution of immune cells at early time points 21. Some studies had also directly explored the relationship between transfused stem cells and IR. Liu et al. 22 showed that the count of high-dose CD34^+^ cells was significantly positively correlated with CD3^+^ T, CD4^+^ T and NK IR. Pei et al. 9 showed that the high-dose MNC count was associated with the improvement of IR, especially CD3^+^ T IR. Our results indicated that the number of CD34^+^ cells reinfused significantly affected the early rapid IR of CD3^+^ T cells. For early rapid IR of NK cells, the number of MNC and CD34^+^ cells were both important. Based on previous studies, our study further showed that it was the number of CD34^+^ cells in the number of MNC that promoted IR, especially early rapid IR. That is, increasing the number of MNC may be not sufficient. We need to try to increase the number of CD34^+^ cells reinfused.

In the process of IR, different from the thymus-dependent and non-thymus-dependent pathways of T cells, the reconstitution of B cells usually comes from the differentiation of donor HPC from native to mature 23. GVHD is predominantly mediated by alloreactive donor T cells, but it is associated with significantly poorer B cell reconstitution in both function and number 24. Studies had showed that B cell reconstitution was delayed in patients who underwent aGVHD with donor T cell infiltration and destruction of bone marrow 25, 26. The risk factor of GVHD is the degree of HLA compatibility 14. However, in our univariate analysis, the occurrence of aGVHD was found to affect the early rapid IR of CD19^+^ B cells, but in multivariate analysis, it was HLA-matched that significantly affected the early rapid IR of CD19^+^ B cells. This enables us to predict IR before transplantation without waiting for the occurrence of GVHD, and prevent infection caused by delayed B-cell IR by giving more gamma globulin infusion to HLA-incompatible recipients in advance.

NK cells are the first quantitatively reconstituted population of donor-derived lymphocytes after HSCT, mainly derive from the differentiation and maturation of progenitor cells, and are predominantly immature in the early stage 27. NK cells play an important role in immune response to viral infection before the recovery of adaptive immunity 28. Patients with low NK cell counts on days 30 and 90 after HSCT had poorer OS 29. In the study of children using various hematopoietic stem cell sources, rapid NK IR showed a trend towards decreased mortality 30. Ando et al. 31 studied the IR of 358 adult patients with hematological malignancies. They found that NK cell was an independent predictor of OS and that patients with rapid NK cell reconstitution on day 100 after transplantation had relatively better OS and DFS. NK cells served as the major circulating lymphocytes in the first 3 months after transplantation. Rapid and stable “mature” NK cell reconstitution will lead to better transplant outcomes 32. In our study, we focused on pediatric patients with AA rather than malignant hematological diseases and found that both OS and FFS in CD56^+^ NK cell early rapid IR group were higher than those in non-IR group. Our results provided preliminary confirmation that the predictive value of early rapid IR of NK cells was also applicable to benign hematologic diseases. The reconstituted NK cells have functional defects for more than 6 months after transplantation 33. It is essential to improve the patients' immunity to viral infection and thus improve the patients' OS and FFS.

Early rapid CD3^+^ CD8^+^ T cell IR could predict superior transplantation outcomes. Koehl et al. 34 observed a significantly higher number of survivors among patients whose CD3^+^ CD8^+^ T count reached above the 5th percentile of age-matched normal level in the first year after HSCT compared to patients who never reached. In all adult patients with hematological malignancies, Ando et al. 31 also found that better CD8^+^ T cell reconstitution at a relatively higher level was an independent predictor of OS. We used the specific reconstituted cell count in the early 3 months after transplantation to predict, and achieved good results. Tian et al. 35 found that patients with CD3^+^ CD8^+^ T cell count >375/uL on day 90 after transplantation had lower infection rate and higher LFS and OS. Our study complemented data on benign diseases in children rather than transplant adult patients.

A study by van Roessel et al. 36 included 315 transplant patients and found that early CD4^+^ T cell IR was an independent predictor of OS, EFS and NRM, and that patients with CD4^+^ T cell IR had higher OS than the non-IR group. Kim et al. 7 studied 69 transplant patients and found that the 2-year OS rate of patients with absolute CD4^+^ T cell count >200/uL in the 3rd month after transplantation was better than that of patients with absolute count <200/uL. Chang et al. 37 studied patients with hematological diseases after Haplo-HSCT and found no association between CD4^+^ T cells on day 30 or 90 and survival. No positive results in our study were obtained in the prognostic impact of CD4^+^ T cell IR. We do not think this should be because our definition and assessment of CD4^+^ T cell early rapid IR are different, considering that it may be different because our patient population is children with AA. Apparently, only 26.2% of children recovered more than 200/uL in the 3rd month after transplantation, but 74% of patients in Kim's study 7 reached IR under the same standard. It may be that our underestimation of CD4^+^ T cell IR biased the conclusions.

Compared with other studies, we demonstrated the IR of AA in children after allo-HSCT and its clinical prognostic value. The main limitation of this study is the small sample size of AA children and further studies with larger sample size are necessary to verify. The time points and thresholds to evaluate whether immune cell subsets can be early rapidly reconstituted vary between studies, so further researches are still needed to draw the final conclusions on the predictive value of IR after HSCT. However, our study and several previous studies had demonstrated that the rapidity and degree of IR after transplantation affected the prognosis of patients. Future researches should also focus on the factors affecting IR and search for strategies that will predict or improve IR to further improve the survival rate of patients and transplant efficacy.

## Figures and Tables

**Table 1:** General clinical characteristics of patients with AA

Characteristics of included patients	
	All patients (N =61)
Gender, female, % (n)	52.5% (32)
Median age at HCT, months (range)	90 (14-201)
Diagnosis, % (n)	
—SAA	44.3% (27)
—VSAA	24.6% (15)
—NSAA	31.1% (19)
Graft type, % (n)	
MRD	34.4% (21)
MUD	16.4% (10)
Haplo	49.2% (30)
ABO matched, %(n)	50.8% (31)
Stem cell source, %(n)	
PB	52.5% (32)
BM+PB	47.5% (29)
MNC count (×10^8/kg, mean ± SD)	14.39±0.79
CD34^+^ cell count (×10^6/kg, mean ± SD)	8.60±0.39

Note: Donor cell chimerism rate: six patients had no indicator of donor-recipient chimerism, and only four did not achieve complete donor type at the first post-transplantation measurement. Patients who underwent Haplo-HSCT were received BM+PBSC (one of the children were received PBSC only) and patients who underwent MRD/MUD-HSCT were received PBSC alone.

**Table 2:** Univariate and multifactorial analysis of factors affecting IR

Parameters affecting immune reconstitution				
	Univariate		Multivariate	
Lymphocyte subset	Parameter	P	Parameter	P
CD56^+^ NK	MNC count	0.05	MNC count	0.041
	CD34^+^ cell count	0.033	CD34^+^ cell count	0.024
CD3^+^ T	CD34^+^ cell count	0.022	CD34^+^ cell count	0.015
	Infections before transplantation	0.034	Implantation time of ANC	0.027
	Implantation time of ANC	0.021		
CD4^+^ T	CD34^+^ cell count	0.04		
CD19^+^ B	HLA matched	0.002	HLA matched	0.045
	Receiving MSC	0.038		
	aGVHD	0.016		
	Graft type (MRD/MUD/Haplo)	0.020/0.036/0.009		

## Abbreviations

AA: aplastic anemia
ALG: antilymphocyte globulin
allo-HSCT: allogeneic hematopoietic stem-cell transplantation
ATG: anti-thymocyte globulin
BM: bone marrow
FFS: failure-free survival
G-CSF: granulocyte colony-stimulating factor
GVHD: graft-versus-host disease
Haplo-HSCT: haploidentical HSCT
IR: immune reconstitution
MNC: mononuclear cells
MRD-HSCT: matched related donor HSCT
MSCs: mesenchymal stem cells
MUD-HSCT: matched un-related donor HSCT
OS: overall survival
PBSC: peripheral blood stem cells
